# Progress in Medicine

**Published:** 1898-05-07

**Authors:** 


### Progress in Medicine.

### DISEASES OF THE HEART AND CIRCULATION.

(*Continued from page* 79.)

Medicine is still at a loss for means either to arrest the development or promote the absorption of pericardial dropsy. The fluid must be drawn off by tapping. This is best done just inside the outer left limit of the line of dulness, *i.e.*, well outside the impulse, the fluid gravitating naturally to this position as the lowest and most distensible part of the sac; or it may be effected by puncture at the left costo-xiphoid angle, thrusting the instrument upwards and backwards. In healthy subjects there would be a chance of wounding the liver or peritoneum, but probably none when the pericardium is distended with fluid, and pressing down the diaphragm.

In a discussion on the diagnosis of adherent pericardium, Broadbent[10] says that it is often extremely difficult, and even impossible, for partial adhesions may give rise to neither symptoms nor physical signs, and even adhesions involving the entire surface of the heart may be attended with little or no interference with its functional efficiency, provided that there were no outside adherence of the pericardium to the chest wall. The symptoms to which adherent pericardium gives rise are not all characteristic, and it is only when other causes of these symptoms can be excluded that we may make such a diagnosis. Sometimes the absence of stasis in the pulmonary circulation, when general dropsy is present from systemic venous obstruction, may suggest that the dropsy is due to the right heart being hampered by pericardial adhesions. Each valvular lesion produces its peculiar effects on the circulatory apparatus, and the absence or modification of these in any case may determine a diagnosis. For example, the absence of the usual compensatory hypertrophy of the left venticle in aortic valve disease may be due to adherent pericardium. Similar effects are still more marked in mitral disease.

Definite physical signs of adherent pericardium are of several kinds, and are due to the heart being bound fast to the pericardium, and this to surrounding structures. Thus, there may be imperfect descent of the apex beat during inspiration, and inadequate shifting of the cardiac impulse when the patient is turned on to the other side. A systolic tug of the left false ribs posteriorly, communicated by the diaphragm, may be conspicuous. Another valuable sign is complete arrest of the slight respiratory movements of the abdominal wall in the epigastric triangle. This is due to limitation of the respiratory excursion of the diaphragm, owing to the adhesions of the heart to the latter, and the impossibility of the heart freely moving owing to its fibrous connections. Little assistance in diagnosis is to be obtained from pulsus paradoxus, or from the effect on the veins of the neck. Lees is of opinion that the influence of adherent pericardium on the heart is indirect; it tends to fix and maintain a condition of dilatation.

**Alcoholic Heart Disease**, though first described some years ago by Bollinger, is a subject which has received but little attention in England. Beddard[11] and Ligertwood[12] have recently given an account of its chief features and discussed its pathology. The majority of cases are well-developed, fat men, between the ages of 25 and 50, who have done heavy work and drunk freely, especially of beer. In the earliest stages the patients complain only of shortness of breath on exertion, and perhaps a little discomfort in the precordium. Many suffer several such attacks, from which they readily recover under rest and purgatives, and abstinence from alcohol; others pass in a short time to the more advanced condition, in which there is cyanosis, orthopnœa, nocturnal dyspnœa, and œdema, which may be chiefly of legs. A favourite situation is the external genitals, though generally some other part of the body is affected as well. The size of the heart is as a rule very difficult to ascertain by percussion owing to subcutaneous fat and emphysema. On auscultation no murmur may be present, but the cardiac rhythm is modified so as to produce a bruit de galop or weak fœtal sounds. The pulse is usually not very much accelerated; in bad cases its irregularity is precisely similar to that met with in bad mitral incompetence. The diagnosis is often most difficult to make—the condition has to be differentiated from the other forms of primary myocardial failure, viz., chronic nephritis with secondary heart failure, arteriosclerosis, syphilitic heart failure, adherent pericardium, fatty and fibroid degeneration. Between these it is often difficult to diagnose clinically, and to a large extent we have to depend on the history of the case. Rest and establishment of diuresis frequently lead to recovery without any active treatment directed to the heart. The mortality is stated to be about 13 per cent. in hospital cases. The pathology of the condition is far from clear. Neuritis of the cardiac nerves has been suggested, but there is little or no evidence in its favour. It also seems unlikely that any considerable degeneration of the muscle can occur considering the rapidity with which the majority of cases recover.

[10] Broadbent, Brit. Med. Jour., Jan. 15, 1898. [11] Beddard, Clin. Jour., Dec. 22, 1897. [12] Ligertwood, Med. Chron., Dec., 1897.

